# Embracing a new chapter at *Epilepsia Open*: A message from the incoming Editor‐in‐Chief

**DOI:** 10.1002/epi4.13048

**Published:** 2024-10-01

**Authors:** Merab Kokia

**Affiliations:** ^1^ Epilepsy Center Lund Sweden

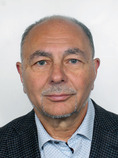

As the incoming Editor‐in‐Chief of *Epilepsia Open*, I am honored to step into this role at a time of significant growth and innovation in epilepsy research. I am excited to lead this distinguished journal, building upon the solid foundation established by my esteemed predecessors. The opportunity to guide *Epilepsia Open* forward in its mission to disseminate high‐quality research is both a privilege and a responsibility that I embrace wholeheartedly.

*Epilepsia Open* was launched in 2016 as the official open‐access journal of the International League Against Epilepsy (ILAE). Over the years, it has rapidly established itself as a reputable platform for rigorous basic, translational, and clinical research in epilepsy. The journal's commitment to transparency and inclusivity in publishing has allowed it to stand out, accepting not only groundbreaking studies but also high‐quality research that reports negative, confirmatory, or preliminary findings ‐ studies that are essential for the robust development of our field.

As I assume the role of Editor‐in‐Chief, I would like to express my deepest gratitude to the outgoing Editor‐in‐Chief, Professor Aristea Galanopoulou, and Deputy Editor, Professor Dong Zhou. Their leadership has been instrumental in shaping the journal's current trajectory. Under their leadership, *Epilepsia Open* has seen a steady increase in submissions, readership, and citations, reflecting the journal's growing influence in the epilepsy research community.

Moreover, I am eager to embrace the opportunities presented by emerging technologies and interdisciplinary research. The field of epilepsy research is evolving rapidly, with new methodologies and big data approaches offering unprecedented insights. I also aim to collaborate with data science and machine learning experts to analyze citation trends and suggest strategies to increase the journal's impact. These advanced analytical tools will not only help us understand the shifting landscape of research influence but also guide us in curating content that resonates with the needs of the epilepsy community.

*Epilepsia Open* is uniquely positioned to serve as a forum for these advancements, and I encourage submissions that push the boundaries of current knowledge and practice. By fostering collaboration across disciplines and leveraging the power of artificial intelligence, we can drive the field of epilepsy research forward, ensuring that our journal remains at the cutting edge of scientific discovery.

In this rapidly evolving landscape of medical research, where technology and innovation are reshaping how we approach epilepsy care, *Epilepsia Open* will continue to be at the forefront. We will actively seek out research that not only advances our understanding of epilepsy but also translates into better clinical outcomes for patients around the world. Our goal is to ensure that every article we publish contributes meaningfully to the ongoing conversation in epilepsy research and care.

I am enthusiastic about working with our dedicated team, including newly appointed Deputy Editor Professor Piero Perucca, associate editors, editorial board members, and peer reviewers. Together, in close collaboration with ILAE, and the Wiley staff, we will strive to maintain the journal's reputation for excellence while exploring new avenues to make our content more accessible and engaging for a diverse, global audience.

As I embark on this new journey with *Epilepsia Open*, I am reminded of the importance of community and collaboration in science. I invite all of you—authors, reviewers, readers, and researchers—to join us in this exciting new chapter. Your continued support and contributions are what make *Epilepsia Open* a thriving and dynamic forum for epilepsy research.

With sincere appreciation,

Merab Kokaia

Editor‐in‐Chief, *Epilepsia Open*

## Data Availability

Data sharing is not applicable to this article as no new data were created or analyzed in this study.

